# Intact reading in patients with profound early visual dysfunction

**DOI:** 10.1016/j.cortex.2013.01.009

**Published:** 2013-10

**Authors:** Keir X.X. Yong, Jason D. Warren, Elizabeth K. Warrington, Sebastian J. Crutch

**Affiliations:** Dementia Research Centre, Department of Neurodegeneration, UCL Institute of Neurology, University College London, UK

**Keywords:** Posterior cortical atrophy (PCA), Alzheimer's disease (AD), Letter-by-letter reading, Pure alexia, Word form dyslexia

## Abstract

Despite substantial neuroscientific evidence for a region of visual cortex dedicated to the processing of written words, many studies continue to reject explanations of letter-by-letter (LBL) reading in terms of impaired word form representations or parallel letter processing in favour of more general deficits of visual function. In the current paper, we demonstrate that whilst LBL reading is often associated with general visual deficits, these deficits are not necessarily sufficient to cause reading impairment and have led to accounts of LBL reading which are based largely on evidence of association rather than causation. We describe two patients with posterior cortical atrophy (PCA) who exhibit remarkably preserved whole word and letter reading despite profound visual dysfunction. Relative to controls, both patients demonstrated impaired performance on tests of early visual, visuoperceptual and visuospatial processing; visual acuity was the only skill preserved in both individuals. By contrast, both patients were able to read aloud words with perfect to near-perfect accuracy. Reading performance was also rapid with no overall significant difference in response latencies relative to age- and education-matched controls. Furthermore, the patients violated a key prediction of general visual accounts of LBL reading – that pre-lexical impairments should result in prominent word length effects; in the two reported patients, evidence for abnormal word length effects was equivocal or absent, and certainly an order of magnitude different to that reported for LBL readers. We argue that general visual accounts cannot explain the pattern of reading data reported, and attribute the preserved reading performance to preserved direct access to intact word form representations and/or parallel letter processing mechanisms. The current data emphasise the need for much clearer evidence of causality when attempting to draw connections between specific aspects of visual processing and different types of acquired peripheral dyslexia.

## 1 Introduction

The concept of the visual word form is one that is well-established within the psychological literature. Cattel (1886) first documented ‘whole word’ reading by demonstrating how briefly presented words were easier to recall than briefly presented meaningless letter strings, and letters have subsequently been shown to be better identified when presented within a word than individually (Reicher, 1969; Wheeler, 1970) or within a non-word (Grainger et al., 2003). More recently, neuroimaging studies have identified an area within the left fusiform gyrus which is specialised for letter and word recognition and which may constitute the visual word form area (VWFA; Cohen et al., 2000). Given the recency of written relative to spoken language as a cultural invention, it is unlikely that a VWFA would have evolved specifically for reading. However, one suggestion is that accumulated reading experience promotes the specialisation of a pre-existing inferotemporal pathway for higher-order visual processing (McCandliss et al., 2003). The current paper emphasises the extent of this functional specialisation by demonstrating remarkably preserved reading in the context of profoundly impaired perception of non-word stimuli.

Neuropsychological evidence supporting the existence of highly-specialised processes for visual word recognition has been derived from patients exhibiting ‘letter-by-letter reading’ (LBL; also referred to as ‘word form dyslexia’ or ‘pure alexia’; e.g., Shallice and Warrington, 1980; Farah and Wallace, 1991; Binder and Mohr, 1992; Warrington and Langdon, 1994; Hanley and Kay, 1996; Cohen et al., 2000). Such patients exhibit intact letter identification and relatively accurate, but slow, reading, whereby response latencies increase in a linear manner proportionate to word length. LBL reading has been suggested to reflect destruction or inaccessibility of a visual word form system, and is associated with damage to the VWFA (Warrington and Shallice, 1980; Cohen et al., 2000).

The attribution of LBL reading to a specific word form deficit has been challenged on two main grounds, namely that the condition and its characteristic word length effects can be accounted for by a general visual deficit and/or a letter identification deficit.

A general visual account of LBL reading suggests that reading, as a complex behaviour, can be disrupted by even the most subtle low-level visual deficits (Friedman and Alexander, 1984; Farah and Wallace, 1991; Price and Devlin, 2003), which propagate by a cascade process to the level of lexical and semantic representations within the visual system (Behrmann et al., 1998a, Behrmann et al., 1998b). A number of single case and case series studies of LBL readers have reported associated impairments on a range of perceptual tasks involving non-orthographic stimuli. For example, Friedman and Alexander (1984) identified an LBL patient who was impaired on tasks of letter identification, object recognition and had an elevated threshold relative to controls in detecting briefly presented pictures. Furthermore, Farah and Wallace’s (1991) patient TU performed poorly on tasks involving the perception of non-orthographic stimuli under time constraints; these results were replicated by Sekuler and Behrmann (1996). More recently, Mycroft et al. (2009) found that seven LBL readers were similarly impaired for both linguistic and non-linguistic stimuli on tasks of visual search and matching, and the LBL group as a whole performed worse than the control group on a task of visual complexity. By contrast, there are documented cases of LBL readers with no discernible impairment in letter identification speed or the identification of rapidly displayed letters (Warrington and Langdon, 2002; Rosazza et al., 2007) or in a range of tasks assessing visual processing, such as complex picture analysis, visual short term memory and picture recognition from unusual views (Warrington and Shallice, 1980). However, proponents of pre-lexical theories of LBL reading tend to dismiss such cases as reflecting insufficiently sensitive assessment of visual processing skills or the use of non-reading tasks which are not making demands comparable to those involved in reading (Behrmann et al., 1998a, Behrmann et al., 1998b; Patterson, 2000).

Alternative accounts attribute LBL reading to an impairment of letter activation. Some accounts suggest that the critical letter processing deficits may be restricted to the identification of individual letters (e.g., Arguin and Bub, 1992, Arguin and Bub, 1993; Reuter-Lorenz and Brunn, 1990; Behrmann and Shallice, 1995). Other accounts ascribe LBL reading to a deficit in the mechanisms responsible for rapid, parallel processing of letters, leading to the less efficient serial encoding of the component letters of a word (Patterson and Kay, 1982; Behrmann et al., 2001; Cohen et al., 2003). One such possible mechanism is the inability to use the optimal spatial frequency band for letter and word recognition, with letter confusability effects emerging at lower spatial frequencies (Fiset et al., 2006). It should also be noted that some authors have argued that deficits in letter processing are common to all LBL readers, while speculating that such deficits may be due to a more basic visual impairment (Behrmann et al., 1998a, Behrmann et al., 1998b).

One observation regarding both the general visual account of LBL reading is that the evidence base is largely associative in nature; that is, most studies claim that the co-occurrence of the characteristics of LBL reading (i.e., accurate but slow reading, with prominent word length effects) and a particular deficit (e.g., impaired perception of non-lexical stimuli) confers support for their chosen position. In addition, proponents of the general visual impairment account have claimed support for their position from control brain-damaged patients who show the complementary association of no perceptual deficit and no impairment of reading (e.g., patient OL; Mycroft et al., 2009). By contrast, in the current study it is argued that such evidence does not prove a causal link between general visual deficits and LBL reading behaviour. This is achieved by presenting evidence from two patients who exhibit profound visual dysfunction in the presence of accurate and rapid word reading. Rather than demonstrating a selective impairment to the visual word form system in the absence of general visual dysfunction, these patients’ reading abilities are remarkably preserved despite grave and diffuse impairments to their visual system.

The two patients reported in this study have a diagnosis of posterior cortical atrophy (PCA), a neurodegenerative condition involving progressive visual impairment in contrast to relatively spared memory functions. The most frequent underlying pathology is Alzheimer’s disease (AD), with PCA patients showing a greater distribution of senile plaques and neurofibrillary tangles in posterior regions of the parietal cortex, the occipital cortex and temporo-occipital junction relative to more anterior cortical areas (Rogelet et al., 1996; Ross et al., 1996; Tang-Wai et al., 2004). Characteristic symptoms of PCA include early visual processing deficits, and disorders of higher-order visuoperceptual and visuospatial processing (Benson et al., 1988; Mendez et al., 2002; Tang-Wai et al., 2004). Reading difficulties are often a prominent feature of PCA, occurring in about 80% of patients (Mendez et al., 2002) and studies on reading ability in PCA have identified a range of deficits, including neglect dyslexia (Mendez and Cherrier, 1998), attentional dyslexia (Saffran and Coslett, 1996), LBL reading (Catricala et al., 2011) and spatial alexia (Crutch and Warrington, 2007).

The main aim of this study was to evaluate the hypothesis that general visual dysfunction necessarily leads to LBL reading. The general visual account predicts that basic visual impairments should be associated with slow, inefficient reading, with prominent word length effects characterised by considerable increases in reading latency with each additional constituent letter. Contrary to these predications, we report two PCA patients who demonstrate highly accurate and rapid reading with equivocal or absent word length effects despite profound visual dysfunction. This preservation of reading skills was observed despite significantly impaired performance on non-lexical chequerboard perception and rapid serial visual letter presentation tasks, failure on which has previously been linked to LBL reading by proponents of the general visual accounts. The reported distinction between intact reading and impoverished visual function raises questions as to whether the evidence cited for general visual accounts of LBL reading truly reflects causation, or merely the association of deficits elicited by damage to contiguous brain regions.

## 2 Methods

### 2.1 Participants

The study participants were two individuals who met current criteria for a diagnosis of PCA owing to probable AD (Mendez et al., 2002; Tang-Wai et al., 2004). This diagnosis was made based on clinical and neuroimaging data, together with the fulfilment of behavioural criteria employed routinely at the Dementia Research Centre. These criteria require an individual to demonstrate episodic memory function above the 5th percentile and at least two out of four scores below the 5th percentile on tests of posterior function, which include the number location and object decision tests from the Visual Object and Space Perception battery (VOSP: Warrington and James, 1991) and graded difficulty tests of arithmetic and spelling (Jackson and Warrington, 1986; Baxter and Warrington, 1994). Written informed consent was obtained using procedures approved by the National Hospital for Neurology and Neurosurgery. The patients were selected for the current study following the observation of visuoperceptual and visuospatial impairment but preserved performance on a screening test for reading (see Table 1).

**Table 1:** Performance on background neuropsychological tests, including verbal memory, word retrieval and comprehension, executive skills, literacy, numeracy and early visual, visuoperceptual and visuospatial processing.

Test	Raw score		Norms/comment
	FOL	CLA	
MMSE^b^	24/30	27/30	FOL: impaired
Short Recognition Memory Test for words^a^, ^c^ (joint auditory/visual presentation)	21/25	24/25	Within normal range
Concrete synonyms test^d^	20/25	24/25	Within normal range
Naming (verbal description)	19/20	11/20	CLA: <1st %ile; FOL: normal limits
Cognitive estimates^e^ (error score)	1	17	CLA: <1st %ile; FOL: normal limits
Calculation (GDA^f^)^a^	0/24	8/24	FOL: <1st %ile; CLA: normal limits
Spelling (GDST^g^ – Set B, first 20 items)^a^	18/20	19/20	Within normal range
Gesture production test^h^	14/15	9/15	–
Digit span (forwards)	11/16 (7 items)	12/16 (7 items)	FOL: 25th–50th %ile; CLA: >50th %ile
Digit span (backwards)	6/16 (3 items)	7/16 (4 items)	Within normal range

*Early visual processing*			
Visual acuity (CORVIST^i^): Snellen	6/9	6/18	CLA: near-normal; FOL: normal
Figure-ground discrimination (VOSP^j^)	17/20	14/20	<5th %ile

Shape discrimination – Efron squares^k^			
Easy (oblong edge ratio 1:1.63)	19/20	20/20	Healthy participants with normal vision
Moderate (oblong edge ratio 1:1.37)	19/20	19/20	Make no errors on difficult version
Difficult (oblong edge ratio 1:1.20)	9/20	14/20	

Hue discrimination (CORVIST)	2/4	2/4	Impaired

*Visuoperceptual processing*			
Object decision (VOSP)^a^	15/20	7/20	CLA: <5th %ile; FOL: 10th–25th %ile
Unusual and usual views^l^: unusual	5/20	0	<1st %ile
Unusual and usual views^l^: usual	18/20	10/20	<1st %ile

*Visuospatial processing*			
Fragmented letters (VOSP)^a^	8/20	0/20	<5th %ile
Number location (VOSP)	5/10	5/10	<1st %ile
Dot counting (VOSP)	7/10	10/10	FOL: <5th %ile; CLA: normal limits
A Cancellation^m^: completion time	60	50	<5th %ile
A Cancellation^m^: number of letters missed	1	0	–

CORVIST reading test	16/16	16/16	–
Graded non-word reading test^n^	24/25	22/25	–

a Behavioural screening tests supportive of PCA diagnosis.

b Folstein et al. (1975).

c Warrington (1996).

d Warrington et al. (1998).

e Shallice and Evans (1978).

f Graded Difficulty Arithmetic test (GDA; Jackson and Warrington, 1986).

g Graded Difficulty Spelling Test (GDST; Baxter and Warrington, 1994).

h Crutch (unpublished).

i James et al. (2001).

j Warrington and James (1991).

k Efron (1968).

l Warrington and James (1988).

m Willison and Warrington (1992).

n Snowling et al. (1996).

**Table 2:** Accuracy and latency data for FOL, CLA and relevant control groups on the word reading experiments.

		Reading skills					
		FOL	Control group	Difference	CLA	Control group	Difference
1. Brown and Ure words	Total correct	72/72 (100%)	71.8/72 ± .4 (99.7% ± .6)	–	72/72 (100%)	72/72 (100%)	–
	Reaction Time (RT)	.60 ± .11	.51 ± .04	*t* = 1.9, *p* = .08	.64 ± .12	.57 ± .06	*t* = 1.2, *p* > .1

2. Coltheart words	Total correct	77/78 (98.7%)	78/78 (100%)	–	78/78 (100%)	78/78 (100%)	–
	RT (regular)	.54 ± .08	.48 ± .04	*t* = 1.2, *p* > .1	.72 ± .34	.53 ± .05	*t* = 10.5, *p* < .001
	RT (irregular)	.59 ± .14	.51 ± .05	*t* = 1.3, *p* > .1	.92 ± .81	.55 ± .05	*t* = 10.5, *p* < .001

3. Schonell words	Total correct	97/100 (97%)	99.3/100 ± 1.0 (99.3% ± 1.0)	*t* = −2.1, *p* = .063	100/100 (100%)	99/100 ± 1.2 (99% ± 1.2)	*t* = 2.8, *p* < .05
	Mean RT	.72 ± .22	.54 ± .07	*t* = 2.2, *p* = .056	.78 ± .31	.60 ± .06	*t* = 2.8, *p* < .05

1. Brown and Ure (1969). 2. Coltheart et al. (1979). 3. Schonell and Goodacre (1971).

**Table 3:** Performance on tests of letter processing.

		Letter identification						Example stimuli
		FOL	Control group	Difference	CLA	Control group	Difference	
Single letter reading	Total correct	20/20 (100%)	20/20 (100%)	–	20/20 (100%)	20/20 (100%)	–	
	Mean RT	.59 ± .09	.48 ± .06	*t* = 1.5, *p* > .1	.82 ± .17	.56 ± .04	*t* = 5.4, *p* < .005	

Temporal masking	Total correct	25/35 (71.4%)	31.5/35 ± .6 (90% ± 1.6)	*t* = −10.1, *p* < .005	22/35 (62.9%)	30.6/35 ± .9 (87.4% ± 2.6)	*t* = −8.8, *p* < .001	
	Recognition threshold	62 msec	16 msec	–	62 msec	22 msec ± 8.8	–	

Rapid identification: letters	150 msec	25/29	28.5/30 ± .60	*t* = −3.9, *p* < .05	25	27.8/30 ± .46	*t* = −5.5, *p* < .005	
	200 msec	28/29	28.25/30 ± .78	*t* = .8, *p* > .2	27	28.2 ± .74	*t* = −1.5, *p* > .1	
	250 msec	28/29	28.25/30 ± .78	*t* = .4, *p* > .3	26	28.8 ± .42	*t* = −6.1, *p* < .005	
	Total correct	82/88 (93.2%)	88/90 ± 1.4 (97.8% ± 1.6)	*t* = −2.7, *p* < .05	78/90 (86.7%)	87.2/90 ± .4 (97.8% ± .5)	*t* = 18.8, *p* < .001	

Rapid identification: numbers	150 msec	13/15	14.75 ± .50	*t* = −3.1, *p* < .05	14/15	14.6/15 ± .89	*t* = −.6, *p* > .2	
	200 msec	14/15	15/15	–	15/15	14.4/15 ± .89	–	
	250 msec	15/15	15/15	–	12/15	14.6/15 ± .89	*t* = −2.6, *p* < .05	
	Total correct	42/45 (93.3%)	44.8/45 ± .5 (99.6% ± 1.1)	*t* = −2.9, *p* < .05	41/45 (91.1%)	43.6/45 ± 2.6 (96.9% ± 5.8)	*t* = −.9, *p* > .2	

Flanked letter identification	Total correct	72/72 (100%)	72/72 (100%)	–	72/72 (100%)	72/72 (100%)	–	
	Mean RT	1.07	.48 ± .12	*t* = 5.3, *p* < .01	1.14	.50 ± .05	*t* = 11.2, *p* < .001	
	Flanker by spacing interaction			*t* = 1.9, *p* = .08			*t* = 7.5, *p* < .001	

**Fig. 1:**
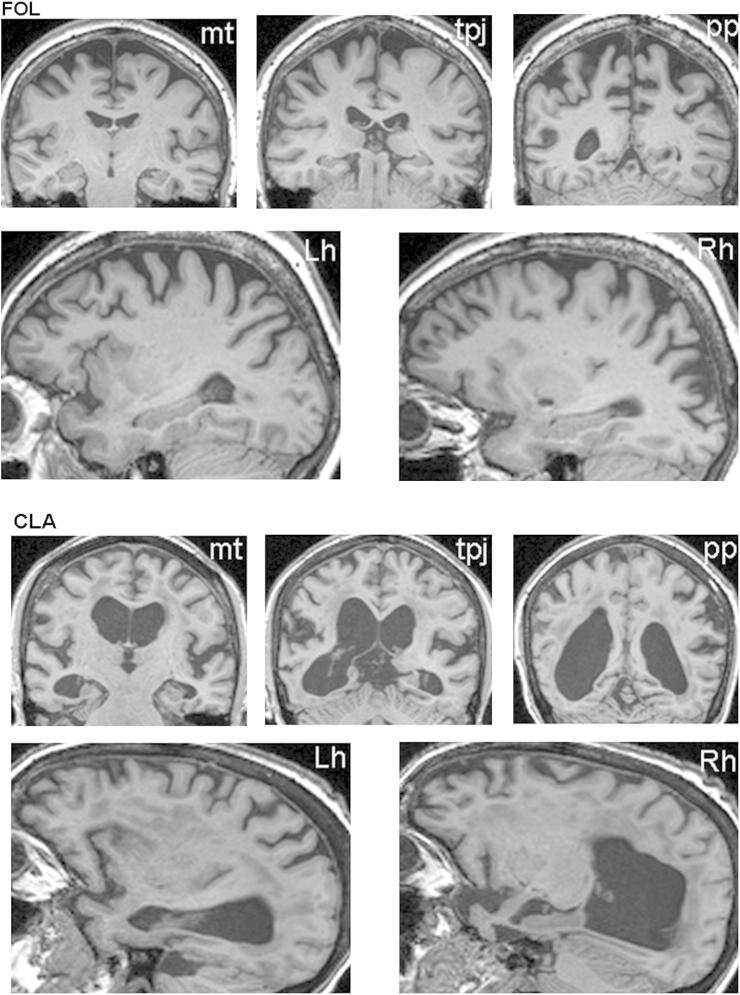
Neuroanatomical features in FOL and CLA. Representative brain MRI sections for each patient show the distribution of atrophy in each case. Coronal sections (upper panels in each case) are in the plane of the mid-temporal lobe (mt), temporo-parietal junction (tpj) and posterior parietal lobe (pp), respectively; the left hemisphere is shown on the right for all coronal sections. Sagittal sections (lower panels in each case) are through the left (Lh) and right (Rh) cerebral hemispheres.

FOL is a 58 year-old right-handed retired administrator for the National Health Service (NHS) who was referred to the Specialist Cognitive Disorders Clinic at the National Hospital of Neurology and Neurosurgery in 2010 with a 4-year history of progressive visual impairment. When seen at clinic she described “looking but not being able to see”, with early symptoms of visual dysfunction including difficulty in locating objects in front of her and problems reading clocks. FOL fulfilled the PCA behavioural criteria (failing tests of arithmetic and spatial and object perception) but her spelling was well preserved. Her memory ability, while not robust, was still within normal limits. Her general neurological examination was normal. Brain magnetic resonance imaging (MRI) (Fig. 1) showed predominantly biparietal atrophy somewhat more marked on the right with relative preservation of the hippocampi, medial temporal lobe structures and no significant vascular burden.

CLA is an 86 year-old right-handed retired classics teacher who was first seen at the National Hospital in January 2011 as part of a clinical assessment. Presenting symptoms included being unable to judge depth and movement and failing to see objects in front of her. CLA fulfilled the PCA criteria, failing tests of spatial and object perception, but spelling and arithmetic were well preserved and she demonstrated strong performance on a test of verbal memory. Her general neurological examination was normal. Brain MRI (Fig. 1) revealed bilateral atrophy of both posterior cerebral hemispheres, more prominent on the right with anterior extension into bilateral peri-Sylvian cortices and the inferior and medial right temporal lobe but relative sparing of the left inferior temporal lobe; additional mild frontal lobe atrophy was evident bilaterally, and there was a mild to moderate degree of small vessel ischaemic damage.

Nine control participants completed all tasks administered to the PCA patients. The controls were split into two groups appropriate for each patient, matched as closely as possible for age, gender and years of education [FOL controls (*N* = 4): mean age 58.4 yrs (range 56–60), all female, mean education: 16 yrs; CLA controls (*N* = 5): mean 83.5 yrs (range 81–84), all female, mean education: 14.8 yrs].

### 2.2 Background neuropsychological data

In addition to the behavioural screening tests, CLA and FOL completed a battery of background neuropsychological tests. Their scores on each task and an estimate of their performance relative to appropriate normative data sets are shown in Table 1. On the Mini Mental State Examination (MMSE), FOL performed below the normal range. She performed well on tests of concrete synonyms, cognitive estimates and naming, and her praxic skills were only mildly impaired to verbal command. She made no errors on a screening test for reading and one error on a non-word reading task.

CLA performed within the normal range on the MMSE. Her concrete synonym comprehension performance was within normal limits but she was impaired on tests of cognitive estimates and naming. CLA had some difficulties on a test of praxic skills, specifically in pantomiming using a toothbrush and hammer. CLA made no errors on a screening test for reading and three errors on a non-word reading task.

### 2.3 Experimental procedures

#### 2.3.1 Visual assessment

Patients FOL and CLA completed a battery of standardised tests examining early visual, visuoperceptual and visuospatial processing:

**Early visual processing**
(i) Visual acuity test from the Cortical Visual Screening Test (CORVIST; James et al., 2001): task required discrimination of squares, circles and triangles at decreasing stimulus sizes corresponding to Snellen form acuity levels.
(ii) Shape detection test from the VOSP(Warrington and James, 1991): figure-ground discrimination task involving random black pattern stimuli (*N* = 20), half with a degraded ‘X’ superimposed. Patients were requested to state whether an “X” was present.
(iii) Shape discrimination: the stimuli (*N* = 60) for this boundary detection task, adapted from Efron (1968), were a square (50 × 50 mm) or an oblong matched for total flux. There were 3 levels of difficulty: oblong edge ratio 1:1.63 (Level I), 1:1.37 (Level II), and 1:1.20 (Level III). The task was to discriminate whether each shape presented was a square or an oblong.
(iv) Hue discrimination (from the CORVIST): the stimuli (*N* = 4) comprised 9 colour patches, 8 of the same hue but varying luminance and one target colour patch of a different hue.

**Visuoperceptual processing**
(i) Object decision (from the VOSP): stimuli (*N* = 20) comprise 4 silhouette images, one of a real object (target) plus 3 non-object distractors.
(ii) Fragmented letters (from the VOSP): participants were asked to identify visually degraded letters (*N* = 20).
(iii) Unusual and usual views (Warrington and James, 1988): participants are asked to identify with photographs of real objects (*N* = 20) pictured from an ‘unusual’, non-canonical perspective. Items not identified from the non-canonical perspective are subsequently re-presented photographed from a more ‘usual’, canonical perspective.

**Visuospatial processing**
(i) Number location (from the VOSP): stimuli (*N* = 10) consist of two squares, the upper square filled with Arabic numerals in different positions, and the lower square with a single black dot. Participants are requested to identify the Arabic numeral whose spatial position corresponds to that of the target dot.
(ii) Dot counting (from the VOSP): stimuli (*N* = 10) are arrays of 5–9 black dots on white background.
(iii) A Cancellation (Willison and Warrington, 1992): participants are requested to mark as quickly as possible with a pencil the location of 19 targets (letter As) presented among distractors (letters B–E) in a grid on an A4 sheet.

**Visuoperceptual/visuospatial processing**
(i) Chequerboard experiment: A set of 24 chequerboard patterns was designed based on an experiment originally developed by Ichikawa (1985) and employed in previous investigations of pure alexia (Mycroft et al., 2009). Chequerboards were composed of either 3 × 3 or 4 × 4 grids with the height/width of individual grid squares being kept constant (subtending .5° of visual angle at a viewing distance of 50 cm). Each chequerboard comprised a pattern of white and black squares, constructed so as to avoid obvious patterns and many squares of the same colour being adjacent to one another (see Table 4). Each chequerboard pattern was paired once with itself and once with another pattern that differed by a single square. This produced a total of 48 pairs, with each pair consisting of chequerboards being presented one above the other at the centre of the screen. Each pair of chequerboards was preceded by a fixation point presented for 1000 msec. Participants were asked to decide whether the chequerboards in each pair were the same or different as quickly and accurately as possible by verbal response. The pairs remained on screen until a response was given and there was a 1000 msec inter-trial interval. One block of 6 practice trials preceded 2 blocks of 24 test trials. Each block contained an equal number of 3 × 3 and 4 × 4 chequerboards.

**Table 4:** Performance on tests of visuoperceptual function.

		Visuoperceptual skills						Example stimuli
		FOL	Control group	Difference	CLA	Control group	Difference	
Chequerboard experiment	Total correct	29/48 (60.4%)	47.3/48 ± .5 (98.4% ± 1.0)	*t* = −32.7, *p* < .001	31/48 (64.6%)	47.6/48 ± .6 (99.2% ± 1.1)	*t* = −27.7, *p* < .001

#### 2.3.2 Word reading

In order to gather a sizeable body of reading responses, all participants were requested to read aloud 3 corpora yielding a total of 250 words. Each corpus was as follows:

1. *Brown and Ure words* (Brown and Ure, 1969): 72 words taken from the Brown and Ure (1969) corpus, which was composed of a subset of words at three levels of length (4, 6 and 8 letters) matched on two levels of frequency and two levels of concreteness.
2. *Schonell reading list* (Schonell and Goodacre, 1971): 100 words of decreasing frequency, ranging in length from 3 to 14 letters.
3. *Coltheart regular/irregular words* (Coltheart et al., 1979): 39 pairs of regular and irregular words ranging from 3 to 8 letters long, matched for word frequency (Kucera and Francis, 1967), concreteness, part of speech and number of letters, syllables and morphemes.

All words were presented in Arial Unicode MS for an unlimited duration within a rectangular fixation box at the centre of the screen; letter height corresponded to a visual angle of 1.2° from a viewing distance of 50 cm.

#### 2.3.3 Single letter processing

A series of letter processing tasks were administered, with all stimuli presented within a central fixation box to ameliorate the effects of visual disorientation:

1. *Letter naming* – all participants were requested to read the letters of the alphabet, excluding I, J, O, Q, W and X, in upper case. Letter height corresponded to a visual angle of 1.2° from a viewing distance of 50 cm.
2. *Rapid serial visual presentation (RSVP) letter/number identification* – letter strings of six letters each were presented serially in the same central spatial position, without an interval between successive letters, as described by previous studies in LBL reading (Warrington and Langdon, 2002; Behrmann and Shallice, 1995). There were three exposure durations of 150, 200 and 250 msec/letter; all participants were tested in nine blocks of 10 strings, with three blocks at each of the three durations arranged in a Latin square design. Before the presentation of each letter string, a target letter was named; participants were asked to decide whether the target letter was present in each string. The target item occurred randomly in positions two to five in each string, with the target item being present in half of all trials. In a subsequent experiment, a similar test was administered using Arabic numeral strings rather than letter strings. The number of trials was halved, resulting in nine blocks of 5 strings.
3. *Flanked letter identification* – all participants were requested to read aloud upper-case letters in 120 trials under the following flanking conditions:
  a. Letters (*N* = 24; e.g., ZNH): alphabetic items excluded the letters I, J, O, Q, W and X, and occurred with equal frequency within each condition (target, left flanker, right flanker).
  b. Shapes (*N* = 24; e.g., ◁N▵): shape flankers consisted of triangles presented at different orientations. The line thickness of targets and distracters was matched.
  c. Numbers (*N* = 24; e.g., 6N5): number flankers consisted of two single digit number flankers chosen from a range between 2 and 9.

In each flanking condition, target letter identification was probed under two spatial conditions, condensed and spaced. The distance between the target letter and flankers was .875 mm in the condensed condition and 8.75 mm in the spaced condition, with the height of stimuli corresponding to a visual angle of 1.0°. The same combination of flankers was used for each target letter under both spatial conditions. The stimuli were presented in blocks of 6 items with the same spacing between the target letter and flankers, with blocks being administered in an ABBA design. All stimuli were presented in the centre of the screen.

### 2.4 Data analysis

Responses were recorded using an Olympus DS-40 digital voice recorder; reading latencies were manually determined from the temporal distance between the onset of audio waveforms corresponding to each stimulus onset and the participant’s spoken response using the digital audio editor Audacity (http://audacity.sourceforge.net). Latency data for erroneous responses and responses where participants had become overtly distracted from the task were removed from the analysis. Analyses of the Brown and Ure (1969) and Schonell (Schonell and Goodacre, 1971) corpora were conducted using multiple linear regression, as neither FOL nor CLA made enough errors to allow the use of a logistic regression model. The regression model was used to relate response latencies to the effects of frequency and length. Overall regression analysis was conducted using a linear mixed model, which was fitted to reaction times with random subject and item effects and fixed effects of length, diagnosis, their interaction and frequency.

Comparisons between both patients and their matched control groups were conducted using a modified *t*-test developed by Crawford and Garthwaite (2002) specifically to identify abnormality of test scores in single case studies. Comparisons between differences in a patient’s scores on two tasks and differences between the control groups’ performance on the same two tasks were conducted the Revised Standardized Difference Test (RSDT) developed by Crawford and Garthwaite (2005). All reported *p* values represent one-way probability.

## 3 Results

### 3.1 Visual assessment

The results of patients FOL and CLA on each early visual, visuoperceptual and visuospatial processing task are shown in Table 1, together with the corresponding normative data. FOL failed every single early visual, visuoperceptual and visuospatial task administered except for visual acuity. On the chequerboard experiment, FOL exhibited significantly poorer performance than controls (*t* = −32.7, *p* < .001) on 3 × 3 and 4 × 4 chequerboards (15/24 and 14/24, respectively) and disproportionately identified chequerboards as being the same (96%) rather than different (25%) (d prime score = 1.057).

CLA was also impaired on all tests of early visual processing except for only mild weakness on a test of visual acuity. She was also impaired on all visuoperceptual tasks and all but one visuospatial task (dot counting). On the chequerboard experiment, CLA exhibited significantly poorer performance than controls (*t* = −27.7, *p* < .001) on 3 × 3 and 4 × 4 chequerboards (16/24 and 15/24, respectively) and was more likely to identify chequerboards as being the same (71%) rather than different (58.5%) (d prime score = .759).

### 3.2 Word reading

The total (and percentage) correct responses and mean (and Standard Deviation (SD)) reading latency data for word reading performance by FOL, CLA and their relevant control samples are shown in Table 2.

1. *Brown and Ure words* – FOL made no error responses, while her control group made one error overall. There was no significant difference between FOL’s response latencies and those of the control group. Regression analysis found a significant effect of length (*t* = 2.2, *p* < .05), but not of frequency (*t* = −.89, *p* > .3) or concreteness (*t* = −1.54, *p* > .1) on FOL’s response latencies. When examining control responses at the group level, neither frequency nor length was significantly related to response latencies, although length was related to response latencies in one individual control.
  Neither CLA nor her control group made any error responses. There was no significant difference between CLA and her control group’s response latencies. Regression analysis found no significant effects of length, frequency or concreteness on the response latencies of CLA or her controls.
2. *Schonell reading list* – FOL made three error responses; two of these were regularisation errors (colonel, homonym), with the remaining error being a visually-based neologism (ineradicable → inerascible). The control group overall made three errors. FOL showed a trend towards being less accurate and having longer latencies relative to controls; however, neither of these effects reached formal levels of significance. Regression analysis found a significant effect of length but not of frequency on response latencies for FOL (*t* = 4.01, *p* < .001) and at the group level for her matched controls (*t* = 4.18, *p* < .001).
  CLA again made no error responses; the control group made a total of five errors between 3 participants. There was no significant difference in response accuracy between CLA and her control group. When examining response latencies, CLA was significantly slower than controls. Regression analysis found a significant effect of length but not of frequency on response latencies for both CLA (*t* = 2.11, *p* < .05) and, at the group level, her matched controls (*t* = 5.4, *p* < .001).
3. *Coltheart regular/irregular words* – FOL made only one visual error response reading irregular words (GAUGE → GAUCHE). The control group made no errors; consequently it was not possible to use a modified *t*-test for error analysis. There was no significant difference between FOL and her control group in the size of regularity effect (RSDT: *t* = .4, *p* > .4).
  Neither CLA nor the control group made any errors. CLA’s response latencies were significantly longer than those of controls for both regular and irregular words. The RSDT identified CLA as being significantly slower for irregular than regular words relative to her control group (*t* = 5.1, *p* < .005).

**Fig. 2:**
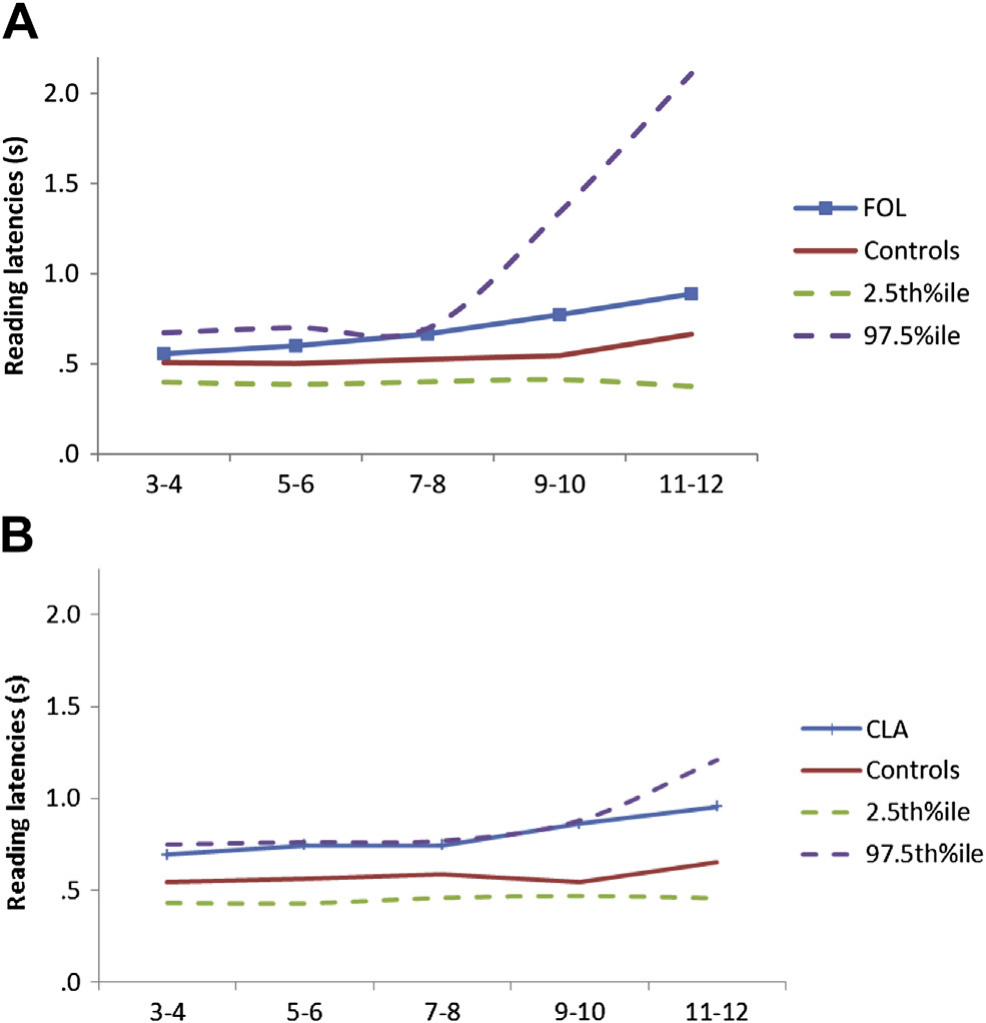
Mean reading latencies for words of different length across all corpora for (A) patient FOL and her matched controls, and (B) patient CLA and her matched controls, with estimated upper and lower control confidence intervals.

*Overall reaction time and word length analysis* – reading latencies for words of up to 12 letters, summing across the 3 reading corpora, are shown in Fig. 2. When examining the response latencies of FOL and her control group, there was a main effect of length (*z* = 2.5, *p* < .05) but not diagnosis (*p* > .3). There was a significant interaction between diagnosis and length (*z* = 2.3, *p* < .05). However, there was significant variation in the size of word length effect within the control group; this was demonstrated by fitting the same model to the control data, plus a second model extended to allow length effects to vary by control participant. Comparison of the two models by a likelihood ratio test identified a highly significant difference in length effects between controls (*p* < .0001).

When examining reading latencies of CLA and her control group, there was a main effect of length on reading latencies (*z* = 3.1, *p* < .005), but only a trend towards a main effect of diagnosis (*z* = 1.9, *p* = .06). There was no interaction between diagnosis and length (*p* > .2).

### 3.3 Single letter processing

The total (and percentage) correct responses and mean (and SD) latency data for letter processing performance by FOL, CLA and their relevant control samples are shown in Table 3.

**Fig. 3:**
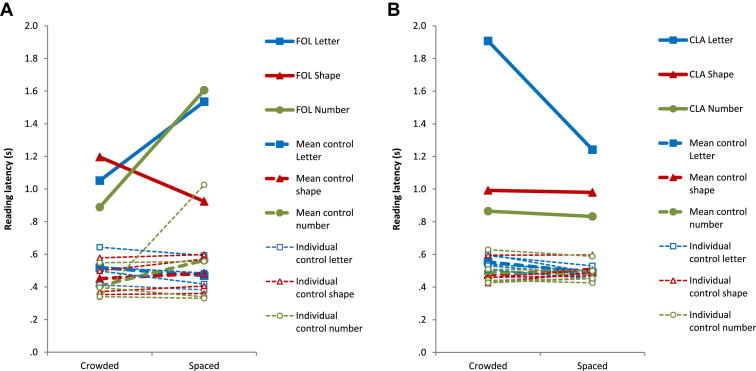
Mean response latencies for target letters under different flanking conditions (letter, shape and number) and spatial conditions (crowded and spaced) for (A) patient FOL and her matched controls, and (B) patient CLA and her matched controls.

1. *Letter naming* – neither FOL nor her control group made any error responses. There was no significant difference between FOL’s reading latencies and those of her control group. Neither CLA nor her control group made any error responses. However, CLA was significantly slower than her control group.
2. *Rapid letter/number identification: letters* – overall letter identification was significantly lower for FOL than her controls; this overall effect reflected significantly lower performance when stimuli were presented for 150 msec but not 200 or 250 msec. CLA also made significantly more errors overall, and specifically when stimulus duration was 150 msec or 250 msec but not 200 msec. *Numbers* – overall, FOL scored significantly lower than her control group. This difference was significant for numbers being displayed for 150 msec, but ceiling effects in the other temporal conditions prevented analysis using a modified *t*-test. There was no significant difference between CLA and her controls for stimuli at any of the tested exposure durations.
3. *Flanked letter identification* – see Fig. 3 for FOL and CLA’s reading latencies. Neither FOL nor her control group made any errors on the flanked letter identification tasks. Summing across all conditions, FOL was slower than her control group. Target–flanker spacing had a significant effect on response latency in only one flanker condition, where target letters were read slower with spaced than condensed number flankers (*z* = −2.2, *p* < .05). There was a trend towards there being an interaction between flanker condition and spatial condition (*t* = 1.9, *p* = .08). As with FOL, neither CLA nor her control group made any errors. Summing across all conditions, CLA was slower than her control group. Target–flanker spacing had a significant effect upon response latency in one flanker condition, where target letters were read slower with condensed than in spaced letter flankers (*z* = 2.0, *p* < .05). There was also one main effect of flanker type, with CLA’s responses in the letter flanker condition significantly slower than in the number flanker condition (*z* = 2.5, *p* < .05). Overall, there was a significant interaction between the group × spacing condition, with target letters being read more slowly with condensed rather than spaced flankers relative to controls (*t* = 7.5, *p* < .001).

## 4 General discussion

The current paper describes two PCA patients, FOL and CLA, who demonstrate preserved reading ability in spite of profoundly impaired visual function. Both patients were impaired on neuropsychological tests of early visual, visuoperceptual and visuospatial processing. Despite these grave visual impairments, both patients were able to read aloud words with perfect to near-perfect accuracy. Reading performance was also rapid, with FOL’s latencies not significantly different to controls on any of the 3 tests of reading, and CLA significantly slower on 2/3 sets but showing only a trend to slower reading overall once frequency was taken into account. In addition, word length effects were equivocal or absent, with FOL showing a modestly increased length effect relative to controls (amongst whom effects of length upon reading latency were also evident) and CLA showing no increase in word length effect. In further contrast to their gravely impaired visual processing, at the single letter level there was only minimal evidence of impaired processing, with patient CLA showing slow (but accurate) single letter identification under normal viewing conditions.

**Fig. 4:**
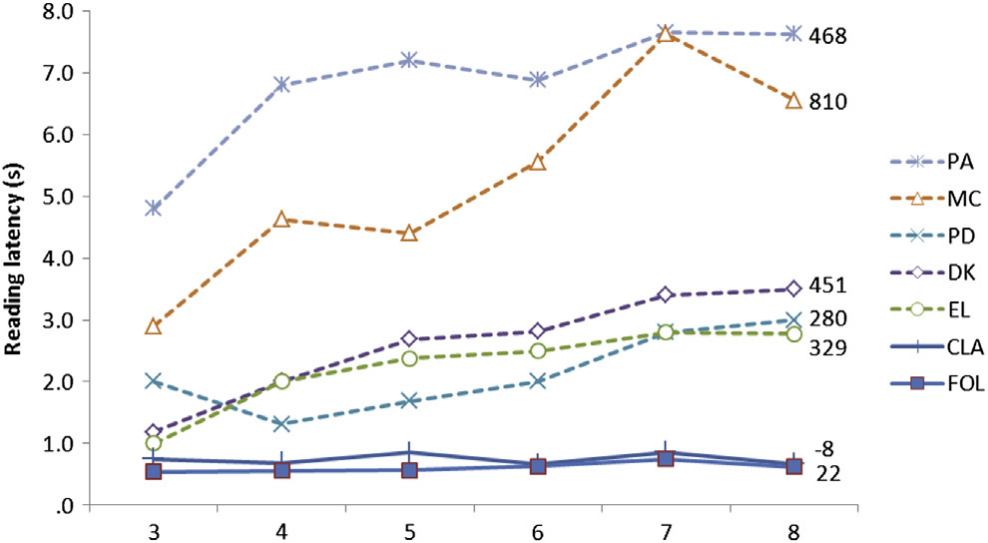
Mean reading latencies for words of different length compared to 5 example LBL readers reported by Mycroft et al. (2009).

Considering each patient’s performance in more detail, FOL’s results seem to indicate her reading ability is almost entirely spared. In each reading corpus, FOL did not differ from her control group in either accuracy or reading latency. Regression analyses conducted on all 250 reading responses (summing across tasks A1, A2 and A3) did reveal a diagnosis (FOL *vs* controls) × length (number of letters) interaction. However, the same analyses found effects of length on reading latencies within matched controls, and length has been shown previously to influence reading speed in normal readers (O’Regan and Jacobs, 1992; Spieler and Balota, 1997). More importantly, the absolute increase in mean reading latency for each additional letter as estimated from the regression model was 36 msec/letter, a small increase which is comparable to that of controls (control mean: 13 msec/letter; control 4: 32 msec/letter) and an order of magnitude different to the increases of 90–7000 msec per additional letter reported in previous descriptions of LBL reading (e.g., Fiset et al., 2005; McCarthy and Warrington, 1990; Mycroft et al., 2009; see Fig. 4). It should also be noted that the trend towards a difference between FOL and the control group’s reading latencies for the Schonell reading test may reflect the particularly low frequency of various words in this corpus (‘somnambulist’, ‘ineradicable’) and FOL’s marginally lower educational level.

The reading accuracy of patient CLA was also excellent, with not a single error recorded on any of the reading corpora. For example, her faultless performance on the demanding Schonell reading test conveys an estimated Intelligence Quotient (IQ) of at least 118 (Nelson and McKenna, 1975). Her reading latencies did not differ from controls on the Brown and Ure words (A1), but reading speed did fall below that of controls on the Coltheart and Schonell tests (A2 and A3), with a significant regularity effect (irregular words slower than regular words) on the Coltheart set. Despite this, the overall difference in latencies across all 250 words failed to reach formal levels of significance. There was also no significant difference between CLA and her controls in the effect of increasing word length.

The main aim of the current paper was to evaluate the claim that general visual dysfunction can account for the acquired peripheral dyslexic syndrome known as LBL reading. General visual function accounts propose that even minor low-level perceptual deficits propagate to or limit activation of lexical representations, ultimately resulting in impaired reading behaviour. One specific prediction of such accounts is that pronounced word length effects are an inevitable consequence of deficits in general pre-lexical processing (e.g., Farah and Wallace, 1991; Behrmann et al., 1998a, Behrmann et al., 1998b; Mycroft et al., 2009). The data presented in the current study fail to support this prediction. Apart from demonstrating accurate and, particularly in the case of FOL, rapid word reading, word length effects were equivocal (FOL) or absent (CLA). This was despite the inclusion of very long words (up to 14 letters) which should maximise any chance of eliciting abnormal word length effects. This failure to detect the dramatic word length effects routinely observed in LBL readers cannot be attributed to preserved visual function, as both patients exhibited dramatic impairments on a wide variety of perceptual tasks. These included a chequerboard task previously used to support the claim that LBL readers have a perceptual impairment that extends beyond alphanumeric stimuli (Mycroft et al., 2009, Experiment 1). However, in asserting that such general visual accounts of LBL reading are incompatible with the data presented here for FOL and CLA, we would wish to state unambiguously that we are not denying that some forms of visual impairment may have an inevitable cost for reading function. Rather we would argue against (i) the pejorative and under-specified use of terms such as ‘general visual impairment’, and (ii) the assumption that *any* form of visual impairment can cause reading impairment. We have previously proposed that visual crowding (the excessive integration of visual features, sometimes referred to as lateral masking) may be one of several specific visual deficits which can cause a particular form of dyslexia (Crutch and Warrington, 2007, Crutch and Warrington, 2009). Indeed, we predicted that any patient demonstrating visual crowding on flanked letter identification tasks would also show some form of visual dyslexia. In line with this prediction, neither FOL nor CLA (whose reading is largely preserved) showed crowding; CLA did show slowed target letter identification particularly with condensed rather than spaced flankers (Task B4), but unlike visual crowding, this flanking effect was only present for flankers of the same category (letter flankers but not number or shape flankers). Given the degenerative nature of the PCA syndrome, we would predict that FOL and CLA’s reading skills will eventually become affected; the task going forward will be to identify any components of visual dysfunction that play a causative role in this predicted deterioration.

The other aim of the paper was to evaluate the hypothesis that impaired letter processing plays a causal role in LBL reading. Such accounts posit that whole reading requires fast parallel letter identification, and that deficits in letter processing inevitably give rise to reading dysfunction and word length effects (e.g., Bub et al., 1989; Howard, 1991; Behrmann and Shallice, 1995; Hanley and Kay, 1996; Price and Devlin, 2003). While both FOL and CLA were significantly less accurate than controls at identifying rapidly serially presented single letters, it is likely that this performance reflects a combination of their basic visual deficits rather than a specific problem of letter processing, particularly as FOL also demonstrated poorer accuracy on an equivalent task looking at rapidly presented numbers. The absence of strong evidence of a deficit in single letter processing suggests that intact parallel letter identification may account for their preserved reading in both patients.

To adequately counter the general visual processing difficulties position it needs to be shown that any visual processing difficulty of the patients shown on some other perceptual task plausibly arises from impairment to a processing system necessary for word reading and not some potentially unrelated visual process. Naturally this is a very difficult point to disprove absolutely. However on these grounds one can make the extremely strong statement that none of the component visual processes required for normal performance on any of the 10 visual tasks evaluated in this study (which examine different levels of the visual system and involve different levels of task difficulty: figure-ground discrimination, shape discrimination, hue discrimination, number location, dot counting, object decision, fragmented letters, canonical and non-canonical view perception, grid experiment), are necessary for intact reading because our patients failed every single task. Furthermore, the impaired processes highlighted by these tasks also do not fall into the poorly-defined category of ‘general visual dysfunction’ which advocates of the general visual account claim cause LBL reading. However, at the much more relative level, the crashing visual deficits highlighted in our patients are an order of magnitude greater than the often subtle deficits claimed for patients cited in support of the general visual account.

Having documented grave visual impairments, it remains to be established what mechanisms support reading in FOL and CLA. The accurate and rapid reading shown by both patients suggests preservation of word form representations or parallel letter processing mechanisms. This notion cannot be verified by the available structural imaging data. However, we note that the MRI scans of FOL and CLA (Fig. 1) both indicate relative preservation of the left fusiform gyrus, commonly cited as the locus of the VWFA (Cohen et al., 2000) and an area in which lesions often result in LBL reading (Binder and Mohr, 1992; Leff et al., 2001; Cohen et al., 2004; McCandliss et al., 2003). This area perhaps provides an anatomical substrate for preserved reading ability in these patients, with one possibility being that strong reading performance is supported by preservation of certain inputs to the VWFA that bypass other impaired aspects of early visual processing. Support for this notion centres on evidence that the VWFA has connections to the primary visual cortex (Rockland and Van Hoesen, 1994; Tanaka, 1997; Haynes et al., 2005) whose relative integrity in FOL and CLA may be indicated by their continued strong or adequate performance on tests of visual acuity. However this suggestion involves the visual word form system maintaining its efficacy, even in the presence of widespread dysfunction at lower levels of the visual system. Irrespective of whether the observed reading is attributable to preservation of the word form and/or aspects of parallel letter processing, the performance of these two PCA patients represents an impressive demonstration of the resilience and efficiency of the reading system in the face of profound visual dysfunction.
