# Corrigendum to “Intact reading in patients with profound early visual dysfunction” [Cortex 49 (9) (2013) 2294–2306]

**DOI:** 10.1016/j.cortex.2016.05.004

**Published:** 2016-08

**Authors:** Keir X.X. Yong, Jason D. Warren, Elizabeth K. Warrington, Sebastian J. Crutch

**Affiliations:** Dementia Research Centre, Department of Neurodegeneration, UCL Institute of Neurology, University College London, UK

The author's would like to acknowledge an error found in Section 2.3.3., point 1 Letter naming. “Letter height corresponded to a visual angle of 1.2°” it should instead read “Letter height corresponded to a visual angle of 1.0°”.

The author's would like to apologise for any inconvenience caused.

